# Toxic and adjuvant effects of silica nanoparticles on ovalbumin-induced allergic airway inflammation in mice

**DOI:** 10.1186/s12931-016-0376-x

**Published:** 2016-05-18

**Authors:** Heejae Han, Yoon Hee Park, Hye Jung Park, Kangtaek Lee, Kiju Um, Jung-Won Park, Jae-Hyun Lee

**Affiliations:** Department of Internal Medicine, Institute of Allergy, Yonsei University College of Medicine, Seoul, Republic of Korea; Department of Internal Medicine, Division of Allergy and Immunology, Yonsei University College of Medicine, Seoul, Republic of Korea; Department of Chemical and Biomolecular Engineering, Yonsei University, Seoul, Republic of Korea; Department of Internal Medicine, Division of Allergy and Immunology, Institute of Allergy, Yonsei University College of Medicine, 50 Yonsei-ro, Seodaemun-gu, 120-752 Seoul, Republic of Korea

**Keywords:** Airway Inflammation, Nanoparticle, Silica, PEGylation, Adjuvant effect, Toxicity

## Abstract

**Background:**

Silica nanoparticles (SNPs) can easily enter in respiratory system via inhalation because of their low molecular weight and ease of dispersion. Toxicity and adverse effects of SNPs vary according to the physical characteristics of the particle.

**Methods:**

To evaluate the toxic and adjuvant effects of 3 types of SNPs in the airway system, six-week-old female BALB/c mice were intranasally administered 3 types of SNPs (spherical [S-SNP], mesoporous [M-SNP], and polyethylene glycol-conjugated [P-SNP]) alone or SNPs/ovalbumin (OVA), three times weekly for 2 weeks. Airway hyper-responsiveness (AHR), bronchoalveolar lavage fluid (BALF), cytokine levels, and histology of the lungs were analyzed.

**Results:**

The S-SNPs/OVA group and M-SNPs/OVA group showed significant AHR, compared to the control group. Among all SNP-treated groups, the group administered SNPs/OVA showed greater inflammatory cell infiltration in BALF, extensive pathological changes, and higher cytokine levels (IL-5, IL-13, IL-1β, and IFN-γ) than those administered SNPs alone or saline/OVA.

**Conclusion:**

Exposure to SNPs alone and SNPs/OVA induced toxicity in the respiratory system. SNPs alone showed significant toxic effects on the airway system. Meanwhile, SNPs/OVA exerted adjuvant effects to OVA of inducing allergic airway inflammation. In particular, M-SNPs showed the most severe airway inflammation in both direct toxicity and adjuvant effect assays. P-SNPs induced less inflammation than the other types of SNPs in both models.

## Background

Silica nanoparticles (SNPs) are among the most common components found naturally in the earth’s crust [1]. SNPs are used in many engineering and medical fields including fabrication, plastics, insulation materials, drug delivery systems, cosmetics, food packing, and coating processes [2]. SNPs are usually believed to be non-cytotoxic and safe. Although SNPs have great importance in nanotechnology, they also have a potential toxic effect, resulting in a health problem. SNPs may both stimulate and suppress the mouse and human immune system and cause injury to cells of several organs [3–6]. SNPs are thought to have different toxic effects depending on their surface characteristics [7]. Many types of SNPs have been developed and studied; spherical type (S-SNP) is a standard form, mesoporous type (M-SNP) has an extremely large surface area, and PEGylated type (P-SNP) has modified surface with polyethylene glycol (PEG). The contact and surface area of M-SNPs is the largest among the three kinds of SNPs (M-SNPs, 70.6 m^2^/g; S-SNPs and P-SNPs, 12.7 m^2^/g). A recent report suggested that the high surface area of SNPs may be useful for drug delivery but also aggravate airway inflammation in murine model and may have adverse effects on human health [8, 9].

Asthma is a chronic airway allergic disease characterized by airway hyper-responsiveness (AHR) and airway inflammation [10]. The prevalence of asthma has been rising recently in urban centers within industrialization regions of Africa, Latin America and Asia [11]. This increase in the prevalence of asthma is thought to be induced by environmental contributors such as air pollutants and nanomaterials [12, 13]. Due to their small size, SNPs can easily enter the respiratory system via inhalation. Exposure to SNPs in manufacturing, managing, and packing through the use and discarding of SNPs may aggravate respiratory diseases, such as asthma, allergic rhinitis, and bronchitis [14]. However, it is not well known whether SNPs exacerbate asthma in asthmatic mouse model or induce adjuvant effects. Toxic effects of SNPs in the airway system upon inhalation are seldom investigated and the mechanism remains unclear. In this study, we aimed to evaluate the toxic and adjuvant effects of three types of SNPs in the mouse airway system.

## Methods

### Silica nanoparticle preparation

SNPs of the spherical (S-SNP), mesoporous (M-SNP), and PEGylated (Polyethylene glycol, P-SNPs) types were provided by the Department of Chemical and Biomolecular Engineering, Yonsei University [15]. SNPs were diluted to stock solutions of SNPS as follows (S-SNP: 10.0 mg/ml, M-SNP: 29.5 mg/ml, P-SNP: 9.0 mg/ml) in saline.

### Characterization of SNPs

**Fig. 1:**
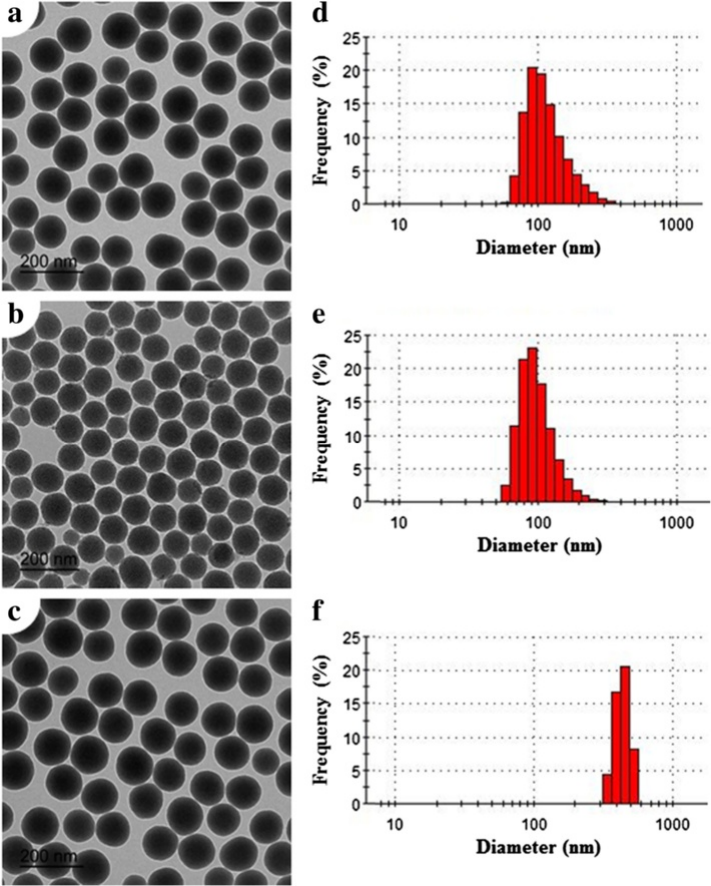
TEM images of SNPs. S-SNPs (**a**), M-SNPs (**b**), and P-SNPs (**c**). DLS analysis of S-SNPs (**d**), M-SNPs (**e**), and P-SNPs (**f**)

Transmission electron microscopy (TEM) was accomplished using a Tecnai 20 to measure the particle size of SNPs (FEI Co., Eindkdk, USA). TEM was performed to the general protocols for TEM study. The particle size of SNPs showed about 100 nm (Fig. 1a–c). Dynamic Light Scattering (DLS; Novato, CA, USA) analysis was used to characterize the size, distribution, and aggregation of the SNPs in diluted saline (Fig. 1d–f). Surface area was measured by Brunauer-Emmett-Teller (BET) equation method. The average pore diameter of the M-SNPs was calculated to be 3.0 nm using the Barrett-Joyner-Halenda (BJH) method.

### Measurement of endotoxin

Limulus Amebocyte Lysate (LAL) assay was used to measure endotoxin levels in SNPs and OVA at concentrations of 1 mg/ml. The LAL assay kit was obtained from Lonza (Walkersville, MD, USA). The detection limit was less than 0.1 EU/ml.

### Animals and treatments

**Fig. 2:**
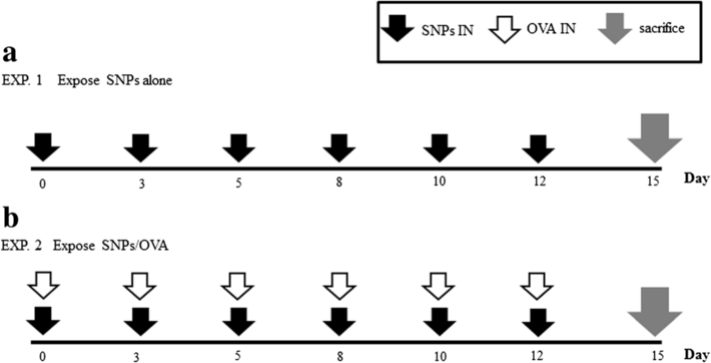
Experimental design of chronic SNPs alone (**a**), and SNPs/OVA (**b**) in mice

Female BALB/c mice, 5–6 weeks old, were purchased (Jungang Animal Experimental, Seoul, Korea). SNPs alone and SNP/OVA model were used 5 mice for each group. All mice were maintained at conventional animal facilities under standard conditions (room temperature of 21–24 °C and relative humidity of 45–70 %, with a 12 h light/dark cycle), and all experimental protocols were approved by the Department of Laboratory Animal Resources, Yonsei Biomedical Research institute, Yonsei University College of Medicine. The present study was approved by the guide for the care and use of laboratory animal guide line. SNPs sized 100 nm were administered via intranasal inoculation of 10 mg/kg per treatment (SNP direct toxicity model, Experiment 1, Fig. 2a) and SNPs 10 mg/kg per treatment and OVA 1 mg/kg (SNP/OVA model, Experiment 2, Fig. 2b) (EndoFit Ovalbumin, invivogen, USA) were administered 6 times over 2 weeks.

### Measurement of airway hyper-responsiveness

Airway hyper-responsiveness (AHR) in response to inhaled aerosolized methacholine was measured using a forced oscillation technique (FlexiVent®; SCIREQ, Montreal, Canada) 48 h after the last challenge. A cannula was inserted into anesthetized mice via tracheostomy, and then the mice were connected to a ventilator. Aerosolized normal saline (0.9 % NaCl) or methacholine (3.125, 6.25, 12.5, 25.0 and 50.0 mg/ml) was administered to the mice for 10 s via a nebulizer. AHR was measured and continuously recorded for up to 2 min.

### Collection of bronchoalveolar lavage fluid

To collect bronchoalveolar lavage fluid (BALF), lungs were lavaged three times with 1 ml of Hank’s Balanced Salt Solution (HBSS, Gibco, USA) through the tracheal tube. The recovered BALF was centrifuged for 3 min at 1,500 g and 4 °C. The whole cells were resuspended in HBSS and the total number of cells was counted using a hemocytometer. BALF cell smears were prepared by cytocentrifugation (Cytospin 3, Thermo, Waltham, USA). The slides were stained, and 100 inflammatory cells including neutrophils, eosinophils, lymphocytes, and macrophages were counted.

### Measurement of cytokine levels

After collecting BAL fluid, the right lung was homogenized using tissue homogenizer (Biospec Products, Bartlesville, USA) in 1.5 ml of RIPA buffer (Thermo, IL, USA) and protease inhibitor solution (Sigma-aldrich). After incubation for 30 min on ice, homogenates were centrifuged at 14,000 x g for 20 min. Supernatants were collected. Concentrations of interleukin-5 (IL-5) (detection limit: 31.2 pg/ml), interleukin-13 (IL-13) (detection limit: 62.5 pg/ml), interleukin-1beta (IL-1β) (detection limit: 15.6 pg/ml), and interferon gamma (IFN-γ) (detection limit: 31.2 pg/ml) in lung homogenate were measured by ELISA (R&D Systems, San Diego, USA) according to the manufacturer’s instructions.

### Histological analysis

After the collection of BALF, the other lung was fixed in 4 % formalin and embedded in paraffin. Lung sections were cut (3 -4 μm) and stained with hematoxylin and eosin (H&E) staining for general examination, periodic acid-Schiff staining (PAS) to measure goblet cell hyperplasia. The number of goblet cells in selected bronchi along the basement membrane was counted on PAS-stained slides at 200× magnification; goblet cell numbers per micrometer of basement membrane were estimated. The slides were observed under light microscopy. Tissue sections were examined with an Olympus BX40 microscope in conjunction with an Olympus U-TV0.63XC digital camera (Olympus BX53F, Center Valley, PA, USA). To determine the percentage of PAS positive epithelial cells, we divided number of PAS-positive by the total cell epithelial cell number. Results are expressed as percentage of PAS-positive cells per bronchiole for each group of mice.

### Statistical analysis

All results are expressed as the mean ± SEM. One-way analysis of variance (ANOVA) was performed using SPSS statistical software version 12.0 (SPSS Inc., Chicago, IL, USA). The AHR data were analyzed with repeated-measures ANOVA followed by a post–hoc Bonferroni test. The other data were analyzed with one-way ANOVA followed by a post-hoc Bonferroni test. *P* < 0.05 was considered statistically significant.

## Results

### Characterization of SNPs

S-SNPs and M-SNPs were spherical and mesoporous in morphology, respectively. The P-SNPs were not different from S-SNPs in terms of morphology. The M-SNPs were globular in shape with multiple pores in each particle, compared to S-SNPs which had no pores (Fig. 1a–c). Measured by the BET equation method, the surface areas of S-SNPs and M-SNP were 12.7 m^2^/g and 70.6 m^2^/g, respectively. The surface area of P-SNPs was similar to that of S-SNPs. In saline, the sizes of M-SNPs and S-SNPs were 100.5 ± 31.35 and 119.6 ± 45.33 nm (Fig. 1d–f), while the size of P-SNPs in saline was 439.1 ± 54.64 nm on DLS analysis. The DLS results showed that the sizes of the three SNPs in D.W. were similar to about 100 nm (Data not shown). Therefore, P-SNPs would aggregate after being diluted in saline, and P-SNP aggregates had significantly larger sizes, compared to other SNPs.

### Detection of endotoxin

The endotoxin levels of all SNPs and OVA were below 0.1 EU/mg.

### Direct toxic effects of SNPs

**Fig. 3:**
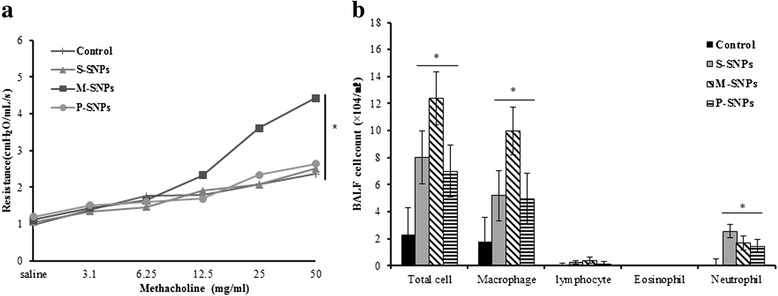
The effect of SNPs alone on the airway inflammation. Values are expressed as means ± SEM (*P < 0.05). Two-way ANOVA showed significant increase for M-SNPs alone exposure on AHR (**a**). Significant increase for SNPs alone exposure on total cell, macrophage and neutrophil in BALF (**b**). (*n* = 5 per group)

**Fig. 4:**
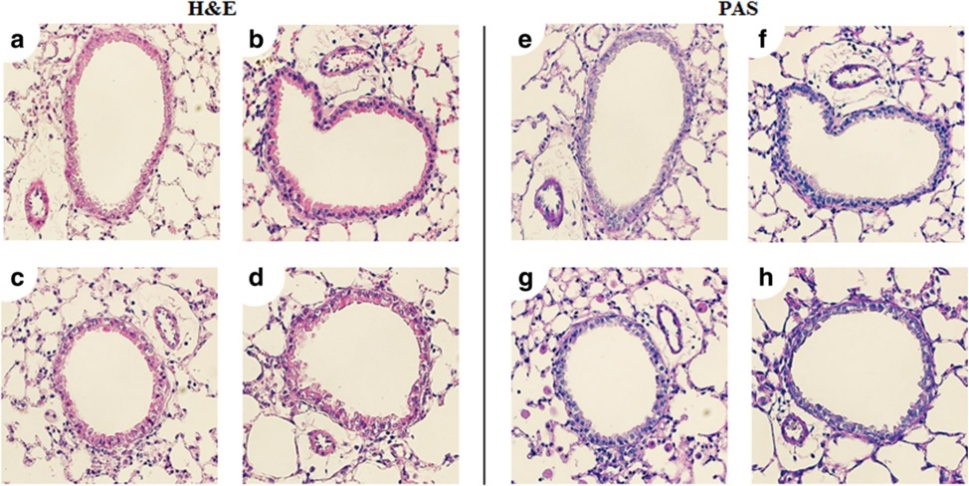
Hematoxylin and eosin stain (H&E stain) and Periodic acid Schiff (PAS) stain was performed on lung sections of mice which exposed SNPs alone. 30 μl saline (**a**, **e**), 200 μg S-SNPs (**b**, **f**), 200 μg M-SNPs (**c**, **g**), 200 μg P-SNPs (**d**, **h**)

Intranasal administration of SNPs alone 3 times per week for 2 weeks induced airway inflammation. M-SNPs induced AHR to a significantly greater extent than any other group. In contrast, S-SNPs and P-SNPs did not induce significant AHR compared to the control group (Fig. 3a). Total cell, macrophage and neutrophil counts in SNP-treated groups were significantly higher than those in control group. Especially, M-SNPs induced more severe inflammation compared to S-SNPs and P-SNPs (Fig. 3b). Histological analysis of lung did not showe peribronchial and perivascular inflammation in all types of SNPs treated groups compared to those in control group (Fig. 4).

**Fig. 5:**
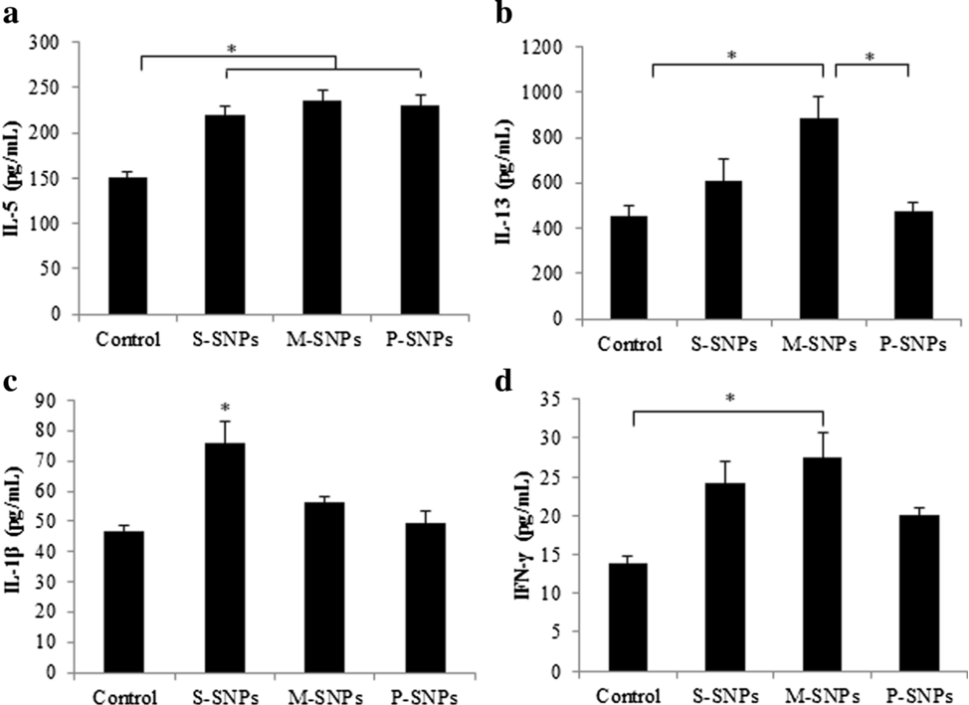
Cytokines were measured from lung homogenates of mice exposed SNPs alone. **a** IL-5, **b** IL-13, **c** IL-1β, **d** IFN-γ. Values are expressed as means ± SEM (*P < 0.05). (*n* = 5 per group)

In analysis of cytokine levels in lung tissues, we observed significant increase of cytokine levels, including IL-5, IL-13, IL-1β, and IFN-γ, in SNPs treated groups. All types of SNPs induced significant increase of IL-5 compared to control group. However, there’s no significant difference among all types of SNPs treated group. The level of IL-13 significantly increased in M-SNPs treated group compared to control group, and even compared to the P-SNP-treated group. S-SNPs and P-SNPs did not induce a significant increase of IL-13 compared to the control group. P-SNPs induced a significantly lower increase of IL-13 compared to M-SNPs. IL-1β levels were significantly increased in the S-SNP-treated group compared to the control group and M-SNP- and P-SNP-treated groups. IFN-γ levels were also significantly increased in the M-SNP-treated group compared to the control group. Although S-SNPs and P-SNPs induced an increase in IFN-γ levels, the difference compared to the control group did not reach statistical significance (*p* = 0.084) (Fig. 5).

### Adjuvant effects of SNPs on allergic sensitization

**Fig. 6:**
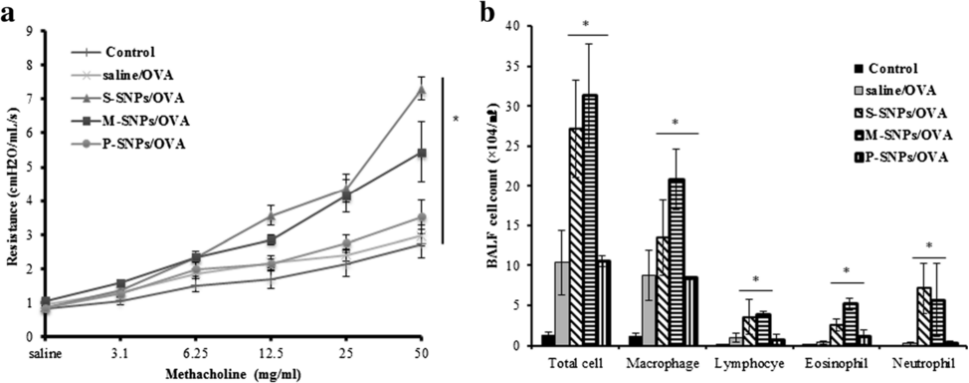
The effect of SNPs/OVA on the airway inflammation. AHR was measured by methacholine in mice exposed SNPs/OVA (**a**). Total cell numbers and number of macrophage, lymphocyte, eosinophil and neutrophil in BALF of mice exposed SNPs/OVA by intranasal (**b**). Values are expressed as means ± SEM (*P < 0.05). (*n* = 5 per group)

Intranasal SNP/OVA administration induced significant airway allergic inflammation. The P-SNP/OVA group showed a similar result to the saline/OVA group. The S-SNP/OVA and M-SNP/OVA groups showed significant AHR compared to the control group and P-SNP/OVA and saline/OVA groups. The P-SNP/OVA group did not show significant AHR compared to the saline/OVA group and control group. The saline/OVA group also did not show AHR compared to the control group. All SNP/OVA-treated groups showed a greater extent of AHR than the SNP-treated group (Fig. 6a).

The BALF analysis results showed the same pattern as the AHR analysis. The number of total cells, macrophages, lymphocytes, eosinophils and neutrophils was significantly increased in the S-SNP/OVA and M-SNP/OVA groups compared to the P-SNP/OVA group, saline/OVA group, and control group. Contrary to the direct toxicity model, M-SNP/OVA and S-SNP/OVA increased the number of not only total cells, macrophages, and neutrophils but also eosinophils and lymphocytes (Fig. 6b).

**Fig. 7:**
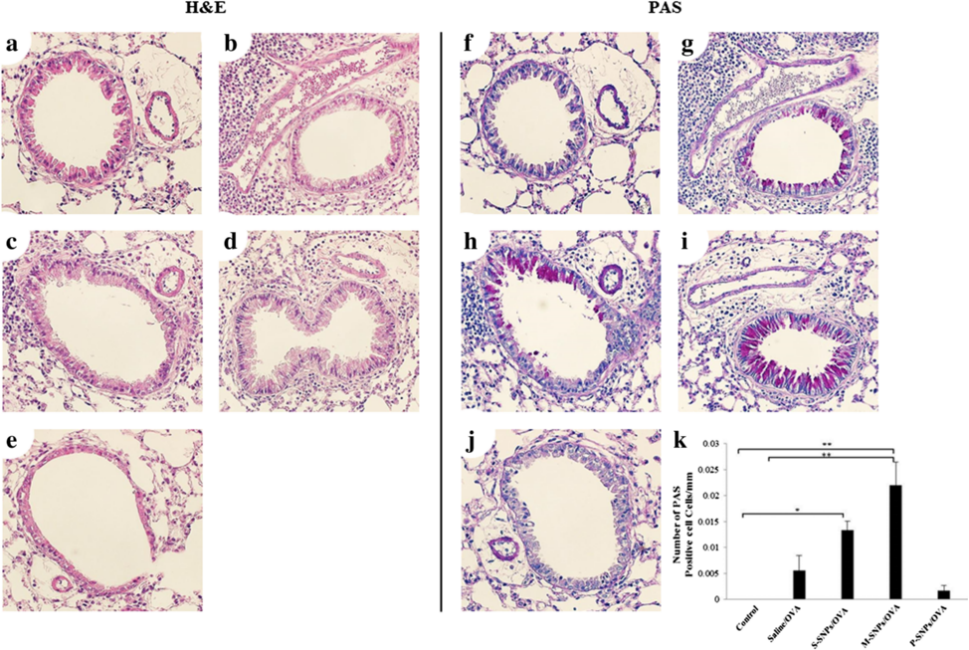
SNPs/OVA group induced airway inflammation compared to control. Hematoxylin and eosin stain (H&E stain) and Periodic acid Schiff (PAS) stain was done on lung sections from mice exposed to SNPs/OVA. 30 μl saline (**a**, **f**), 20 μg saline/OVA (**b**, **g**), 200 μg S-SNPs/OVA (**c**, **h**), 200 μg M-SNPs/OVA (**d**, **i**), 200 μg P-SNPs/OVA (**e**, **j**). Quantitation of inflammation cells was measured by Metamorph system

Histological analysis showed peribronchial and perivascular inflammation in all SNP/OVA-treated groups. In PAS staining, all SNP/OVA groups showed an increase of goblet cell metaplasia. Especially the number of PAS-positive divided by the total cell epithelial cell number was significantly increased in the S-SNP/OVA and M-SNP/OVA group compared to the control group. However, the P–SNP/OVA group presented less inflammation and less increase of goblet cell metaplasia than the other SNP/OVA groups (Fig 7).

**Fig. 8:**
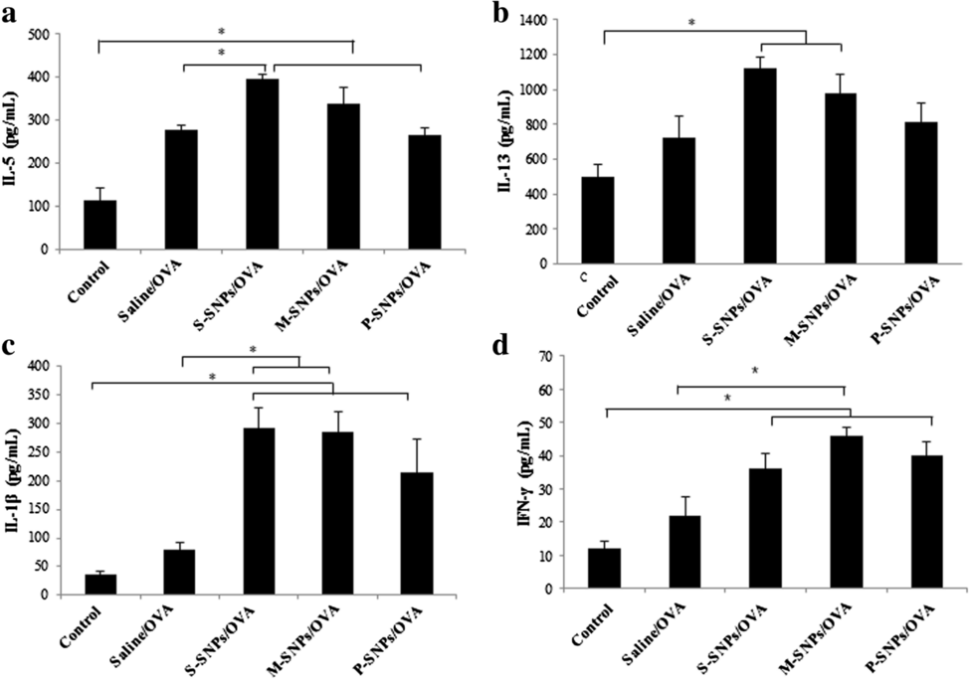
Cytokines were measured from lung homogenates of mice exposed SNPs/OVA. IL-5 (**a**), IL-13 (**b**), IL-1β (**c**), IFN-γ (**d**). Values are expressed as means ± SEM (*P < 0.05). (*n* = 5 per group)

In the SNP/OVA-treated groups, the levels of cytokines, including IL-5, IL-13, IL-1β and IFN-γ, was significantly increased compared to the control group. Particularly in the S-SNP group, the levels of IL-5 and IL-1β were significantly increased compared to the saline/OVA group. In the M-SNP group, the levels of IL-1β and IFN-γ were significantly increased compared to the saline/OVA group (Fig. 8).

## Discussion

SNPs are mainly used in common applications such as cosmetics and packaging, and they have recently used in drug delivery systems. SNPs are believed to be safe for even the human body [16]. However, the effects of exposure to SNPs with or without other allergens on the respiratory system are not well known. In this study, we demonstrated that toxic effects and adjuvant effects of SNPs on the airway system depended on their surface morphology in a murine model. Although this study is similar to the Brabdenberger et al. study in terms of the effects of co-exposure to OVA and SNP on the development of allergic airway disease, we aimed to investigate whether treatment of SNPs as only a challenge without sensitization induces allergic airway inflammation, unlike the Brabdenberger et al. study [14].

In this study, SNPs were administered via six intranasal injections of 200 μg over 2 weeks. The dose of SNPs (200 μg/mouse) was determined on the basis of studies on SNP toxicity in airway inflammation [9, 17]. Furthermore, in our preliminary study, we evaluated the effects of SNPs on airway inflammation by administration of two, four, and six times over 2 weeks. The administration of SNPs for six times over 2 weeks induced airway inflammation, whereas two and four administrations did not (Data not shown). We determined that this dose and frequency of SNP administration is appropriate for visualizing the effects of SNPs on airway inflammation in mice.

Inhalation exposure of SNPs showed toxic effects in the airway system with exacerbation of significant AHR, inflammatory cell increase in the BALF, peribronchial inflammation in histological examination, and increase of cytokine levels in lung tissue. Among all types of SNPs, M-SNPs alone induced statistically significant airway inflammation. The M-SNP-treated group showed significantly increased AHR, inflammatory cell increase in the BALF, and increased levels of IL-5, IL-13, IL-1β, and IFN-γ. Therefore, that M-SNPs induce airway inflammation is likely to be associated with both Th1 immunity (IL-1β and IFN-γ) and Th2 immunity (IL-5 and IL-13), simultaneously [18, 19]. These results showed that M-SNPs could induce airway inflammation, thus leading to remarkable clinical improvement in the respiratory system.

Although M-SNPs induced significant AHR and increased levels of IL-13, it is difficult to say that M-SNPs induced typical asthmatic features in mice. M-SNPs did not induce eosinophilic infiltration in the BALF or pathologic findings. M-SNPs also increased levels of IL-1β and IFN-γ, which are associated with Th1 immunity. We thus assume that M-SNPs induce nonspecific airway inflammation associated with Th1 and Th2 immunity.

Though these results, we suggest that exposure to SNPs alone is not sufficient to induce inflammatory cell infiltration surrounding the bronchial tubes, but it is still clearly toxic.

In the adjuvant effect model, SNPs showed an adjuvant effect in development of allergic airway inflammation by OVA. Intranasal challenge with saline/OVA did not induce allergic airway inflammation, however the SNP/OVA-treated groups showed significant airway inflammation, remarkable AHR, increased inflammatory cell counts in the BALF, perivascular/peribronchial inflammation in histopathology, and increased levels of various cytokines compared to the saline/OVA-treated group. Contrary to the SNP-alone model, the SNP/OVA-treated group presented more significant AHR and inflammatory cell infiltration, including eosinophils. Eosinophil infiltration is a specific finding of typical asthma associated with Th2 immunity [20, 21]. These findings support that SNPs have an adjuvant effect for OVA in the induction of airway inflammation. However, the grade of eosinophilic infiltration in SNP/OVA-treated model was lower than that in a typical asthma model. The levels of Th1-associated cytokines (IL-1β and IFN-γ) also increased in the SNP/OVA-treated group. Although SNPs act as an adjuvant to OVA to induce allergic airway inflammation, their effect is not sufficient to induce pure allergic inflammation in comparison with another typical allergens, such as OVA.

Some molecules such as particulate matter (PM) and pollutants are known to have adjuvant effects for allergic sensitization and to induce allergic inflammation in conditions with exposure to typical allergens [22]. Some patients who are exposed to typical allergens but have no allergic disease could develop allergic diseases if they are exposed to typical allergens in the presence of adjuvants such as PM and pollutants. Because SNPs also have an adjuvant effect for induction of allergic airway inflammation by OVA similar to these molecules, SNPs may induce allergic disease in patients who have no allergic disease in the presence of typical allergens.

In both models of the present study, P-SNPs treatment induced less inflammation. The contact area is a critical factor for inducing toxicity during interaction between SNPs and lung tissue. Large surface area is useful for drug delivery, labeling, and gene delivery [23]. However, a smaller surface area results in less opportunity for tissue interaction and thus less inflammation when SNPs are absorbed via the intranasal route [8, 24, 25]. Therefore, we suggest that the larger surface area of M-SNPs could induce more severe inflammation than that induced by other SNPs.

Although S-SNPs and P-SNPs have a similar diameter and surface area, P-SNPs induced airway inflammation to a lesser extent than S-SNPs. These results may be due to the aggregation characteristic of P-SNPs due to their surface coating; S-SNPs and M-SNPs were dispersed. Some studies have been reported that aggregated forms of nanoparticles show less toxicity than dispersed nanoparticles [26, 27]. In this study, P-SNPs suspended in D.W. were not aggregated, while those suspended in saline were. Therefore, we raise the possibility that P-SNPs are less toxic owing to their surface area and propensity to aggregate. However, further studies are needed to assess toxicity of P-SNPs in relation to aggregation and dispersion patterns in more detail.

In summary, the results of our study indicate that SNPs alone and SNPs with OVA can induce airway inflammation in mice. These findings suggest that exposure to SNPs alone might enhance airway diseases, such as asthma. The toxicity of SNPs may different depending on their coatings and dispersion patterns. We suggest that P-SNPs might be safer than S-SNPs and M-SNPs for medical applications in humans.

## Conclusions

Exposure to SNPs alone and SNPs/OVA resulted in toxicity in the airway system. Our data suggest also that SNPs have adjuvant effect for OVA to induce allergic airway inflammation. Notably, M-SNPs showed the most severe airway inflammation in both the direct toxicity and adjuvant effect assays. P-SNP appeared to be less harmful than other SNPs to the airway system.

## Abbreviations

AHR: airway hyper-responsiveness
BALF: bronchoalveolar lavage fluid
BET: Brunauer-Emmett-Teller
BJH: Barrett-Joyner-Halenda
H&E: hematoxylin and eosin
M-SNPs: mesoporous silica nanoparticles
OVA: ovalbumin
P-SNPs: polyethylene glycol-conjugated silica nanoparticles
PAS: Periodic acid-Schiff staining
S-SNPs: spherical silica nanoparticles
SNPs: silica nanoparticles
TEM: transmission electron microscopy
